# LLM-Storyteller: child-guided narrative co-creation via real-time adaptive prompting and visualization with an embodied robot

**DOI:** 10.3389/frobt.2026.1882463

**Published:** 2026-09-21

**Authors:** Fatimah Alali, Saad Ezzini

**Affiliations:** 1 Information and Computer Science Department, King Fahd University of Petroleum and Minerals, Dhahran, Saudi Arabia; 2 Interdisciplinary Center for Intelligent Manufacturing and Robotics, King Fahd University of Petroleum and Minerals, Dhahran, Saudi Arabia

**Keywords:** child–robot interaction, embodied robotics, human–robot interaction (HRI), large language models (LLMs), narrative co-creation

## Abstract

Interactive storytelling has shown great potential in supporting children’s creativity. However, existing robotic storytelling systems mostly treat the robot as the primary author or lack the ability to adapt to a child’s interaction state in real time. In this paper, we present LLM-Storyteller, an embodied robotic system designed for child-guided narrative co-creation. The system preserves the child’s authorship by acting solely as a facilitator through adaptive prompting, which adjusts based on real-time classification of the child’s interaction state. Furthermore, it closes the creative loop by transforming the child’s narrated story into a personalized animated cartoon. The architecture integrates local automatic speech recognition, state detection, an adaptive Large Language Model (LLM) prompt engine using Llama 3.2, and a story-to-animation pipeline utilizing Stable Diffusion XL. We evaluated the system across 12 simulated storytelling sessions, all of them role-played by the two authors rather than by children, measuring response latency, animation generation time, interaction state distribution, and the lexical origin of the narrative. Authorship was approximated by Lexical Narrative Ownership, the share of unique content words in the finished narrative that the simulated child introduced before the robot did. This share reached 0.87 on average, and 58 of 72 robot turns followed the measured question-based response format. Together, these indicate that the narrative vocabulary originated with the child, which is evidence of facilitation rather than a semantic proof of story ownership. An internal rating of the outputs by the two authors across four dimensions gave the highest scores to follow-up question quality and the lowest to image-story alignment. All findings are bounded to prototype-level feasibility under simulated conditions, since no children took part.

## 1 Introduction

Storytelling is a fundamental activity that supports cognitive development and creative thinking in children. In recent years, social robots have been increasingly utilized to engage children in narrative activities. Early systems relied on rule-based or scripted architectures, which provided limited adaptability to individual children. The emergence of Large Language Models (LLMs) has expanded the possibilities for open-ended child-robot interaction. However, most LLM-driven storytelling robots act as co-creators that generate story content themselves, rather than empowering the child to lead the creative process. Furthermore, existing systems often lack real-time adaptation to the child’s specific interaction states, such as hesitation or short responses, and they do not provide a personalized visual output based on the child’s original story.

To address these limitations, we developed LLM-Storyteller, an embodied robot that supports child-led storytelling and generates personalized animated stories. The system ensures the child remains the primary author while the robot serves as a facilitator through structured, adaptive prompts. By advancing educational technologies through interactive robotic storytelling, this work directly supports the United Nations Sustainable Development Goal 4 (Quality Education), and its development of an advanced edge-computing embodied architecture aligns with SDG 9 (Industry, Innovation and Infrastructure). The study is guided by two research questions.

- RQ1: How can an LLM-powered robot facilitate a child-led storytelling process without overtaking the authorship of the narrative?
- RQ2: What is the technical feasibility and output quality of integrating real-time interaction state detection with an automated story-to-animation pipeline on an edge-computing setup?

Each question is tied to a specific measure. RQ1 is answered by two transcript-derived indicators, Lexical Narrative Ownership and the share of robot turns that followed the measured question-based response format, both defined in Section 4.4. RQ2 is answered by component latency, cartoon generation time, the observed distribution of interaction states, and an internal rating of the outputs on four dimensions. Section 5.4 states each answer next to the evidence behind it. The present evaluation was conducted under simulated conditions without child participants, as detailed in Section 4 and Section 6.

### 1.1 Contributions

The main contributions of this research are:

1. The development of an end-to-end embodied robotic system that supports child-guided narrative co-creation.
2. A real-time interaction state classifier that adapts the robot’s prompts across eight predefined interaction states.
3. An automated story-to-animation pipeline that transforms the child’s spoken narrative into a visual cartoon without relying on cloud-based generation services.
4. A transcript-level measure of narrative authorship that makes the facilitation claim testable rather than rhetorical.

The rest of the paper is organized as follows. Section 2 reviews related work in storytelling systems and visual narratives. Section 3 details the methodology and system architecture. Section 4 describes the evaluation metrics and setup. Section 5 presents the results and discussion of the system’s performance. Sections 6 and 7 outline the limitations, conclusion, and future work.

## 2 Related work

The prior work relevant to this research falls into four areas: pre-LLM robotic storytelling systems, LLM-driven storytelling systems, visual narrative approaches that enable story-to-animation transformation as a creativity support tool, and prior work on interaction states in child-robot dialogue, which informed the definition of the eight interaction states used in the proposed system. Table 1 summarizes the first three.

**TABLE 1:** Summary of related work by category.

Category	Key characteristics	Main limitations	Refs
Pre-LLM storytelling systems	WoZ or scripted robot behavior turn-taking story co-creation emotion-expressive robot actions creativity measured by human raters	No language model No child state adaptation No visual story output	Sun et al., 2017 Elgarf et al. (2022) Antunes et al. (2022) Sandoval et al. (2022)
LLM-driven storytelling systems	GPT/fine-tuned LLM for story generation RL-based non-verbal behavior adaptation classroom or group deployment engagement sensing (gaze, affect)	robot co-authors story content No fine-grained child state model No personalized animated output	Elgarf et al., 2024 Barbato et al. (2025) Tozadore et al. (2025) Tozadore and Rusu (2024)
Visual narrative for creativity support	stop-motion and multimedia animation audio-visual story transformation animation-based creativity assessment	No robot or adaptive dialogue static media, single session No real-time behavior response	Undheim and Hoel, 2023 Zhou et al. (2025) Lin (2026) Atesgoz and Sak (2021)
Proposed system	child as primary author 8-state real-time interaction detection adaptive LLM prompt engine story-to-cartoon pipeline fully local, privacy-preserving	user study with children is future work	—

### 2.1 Pre-LLM storytelling systems

The early storytelling research reveals the potential of establishing embodied co-creative robots; however, it relied on scripted and rule-based architectures, which limited flexibility and adaptability.

A Wizard-of-Oz feasibility study with 78 children aged 4 to 10 compared contextual and non-contextual prompts (Sun et al., 2017). Contextual prompts led to more organized and meaningful stories, while non-contextual prompts sometimes increased how much children spoke at the cost of clarity. Prompt form and timing therefore both matter. A human operated the robot, so the system offered no automatic adaptation.

CreativeBot (Elgarf et al., 2022) crossed robot creativity with collaborative and non-collaborative interaction styles in a 2 × 2 design, with trained raters scoring fluency, flexibility, elaboration, and originality. A creative robot improved children’s creative thinking, while the interaction style made no significant difference. Response quality rather than interaction framing appears to be the more influential factor. The robot’s behavior was pre-designed, and it generated all of the story content.

Robot expressiveness also influences storytelling interaction. In Antunes et al. (2022), an emotionally expressive robot used in a multi-sensory storytelling activity encouraged children with and without visual impairments to include more emotional content and accept one another’s ideas more readily. The study in Sandoval et al. (2022) similarly found, through a storytelling activity with adults, that robot motion and perceived human-likeness influenced judgments of the robot’s creativity. Together, these findings highlight the role of embodied expression in creative human-robot interaction.

Beyond creativity, robotic storytelling companions have also been shown to support language development in preschool children (Kory and Breazeal, 2014) and to shape reading engagement in the home (Michaelis and Mutlu, 2018). Both systems narrate or co-read rather than hand narrative control to the child, which is the gap the present work targets.

### 2.2 LLM-driven storytelling systems

The integration of LLMs into social robots substantially expanded the space of possible child-robot storytelling interactions by enabling contextually coherent, open-ended responses that rule-based systems cannot produce.

The authors Elgarf et al. (2024) conducted a study that combined a Wizard-of-Oz setup with an autonomous LLM system based on a GPT model trained on children’s storytelling data to compare creative and non-creative robot behavior. The results confirmed the earlier findings from Elgarf et al. (2022) and demonstrated that an LLM-powered creative robot improved children’s creativity on the same fluency, flexibility, and elaboration indicators, with the addition of relevant ideas during the story identified as the main driver. The system followed a speech recognition-to-LLM-to-speech generation pipeline.

Building on this, the study Barbato et al. (2025), developed a system that combines an LLM-based story generator with Reinforcement Learning to control the robot’s non-verbal behavior. This includes voice pitch, animation level, and face tracking, which are adjusted based on user signals such as gaze, facial expressions, and body posture. The system was tested with adult participants. The results showed that the adaptive version, which learns and adjusts its behavior, was perceived as more socially intelligent and maintained user engagement better than a version with random behavior. This demonstrates that automatically adapting robot behavior based on real-time user signals can improve the quality of interaction.

The work presented in Tozadore et al. (2025), Tozadore and Rusu (2024) tested LLM-based storytelling robots in real classroom settings. The authors in Tozadore et al. (2025), deployed a robot powered by ChatGPT-3.5 called RoboBuddy in primary school classes for multilingual children. The system generates stories related to the curriculum and includes comprehension questions. Feedback from teachers and children’s drawings showed that students enjoyed the experience and interacted well socially. The study by Tozadore and Rusu (2024) followed a similar approach in maker-space robotics classes. Teachers entered lesson content through a web application, which converted it into prompts for GPT-3.5, and the robot narrated the resulting stories. Teacher interviews informed the system design, followed by testing in classroom sessions. As with RoboBuddy, the robot delivers content generated for the child rather than eliciting the story from the child turn by turn.

### 2.3 Visual narrative and animation for creativity support

Empirical studies have demonstrated the effectiveness of visual narratives and animation in supporting automated story transformation as a creativity enhancement mechanism. A qualitative case study conducted in Undheim and Hoel (2023) involved six kindergarten children and a teacher collaboratively producing a stop-motion animated story across multiple sessions. The results showed that the use of a social-semiotic multimodal framework, which integrates image, sound, and movement through iterative production, deepened narrative understanding and sustained creative engagement. Similarly, the work in Zhou et al. (2025) examined the effect of multimedia transformation more quantitatively. The findings suggest that combining sound and animation as a real-time transformation of children’s drawings significantly increased positive valence, arousal, and narrative creativity scores, specifically idea quantity and idea elaboration, compared to a control condition.

The authors in Atesgoz and Sak (2021) developed a test called TOSCAC to measure creativity in children aged 4–7 using short animated scenes. Their results showed that animation is a reliable and effective way to evaluate children’s creative thinking. In addition, the study Lin (2026) proposed the Interaction Hyperspace Framework to support storytelling in animation education. The framework identifies eleven key elements, such as actions, consequences, and timing of events, that help guide children in building creative and structured stories. Animation has also been linked to gains in story-writing skill among primary school students Al azzam and Khodair, 2025, and AI-generated cartoon imagery has been used as a stimulus for indirect creativity indicators in classroom settings (Zb and Khan, 2024). What these approaches share is that the visual artifact is produced for the learner rather than from the learner’s own spoken narrative.

### 2.4 Interaction states in child-robot dialogue

The eight states used by our prompt engine are not proposed as a new taxonomy of child behavior. Rather, they constitute an engineering abstraction for selecting a prompting strategy from observable features of the interaction transcript. Their overall design is informed by scaffolding theory, according to which assistance should be adapted to the learner’s current performance and gradually reduced as the learner becomes able to proceed more independently (Wood et al., 1976; Vygotsky, 1978). In our system, silence and brief responses are treated as possible indications that additional prompting may be helpful, motivating encouragement or elaboration rather than the introduction of substantial new story content. We do not interpret these cues as direct measures of the child’s competence.

Filled pauses such as um and uh have been associated with anticipated delays in speech production, including word search and deciding what to say next (Clark and Fox Tree, 2002). This motivates the hesitation state and its simplified imagination prompt. Prior child-robot interaction research has shown that observable engagement cues can be used to determine when a robot should apply a re-engagement strategy (Leite et al., 2015). Following this general principle, our system treats repeated content as a possible indication that the narrative has stalled. It also includes an excitement state intended to sustain highly engaged interaction, although its implemented detector relies on orthographic indicators rather than direct measures of vocal enthusiasm. These interpretations are engineering operationalization rather than categories adopted directly from that study. Finally, the prompt-timing result of Sun et al. (2017) indicates that both the form and timing of a robot’s prompt can influence children’s storytelling contributions.

The mapping from these general findings to the eight system states, and from each state to a detection rule and robot strategy, is given in Table 2. We make no claim that the states constitute an exhaustive psychological model of child behavior or that their thresholds are optimal. They are transparent, inexpensive to compute on-device, and provide a practical mechanism for implementing adaptive prompting. Establishing their behavioral validity, evaluating all states with positive examples, and tuning their thresholds using real child speech remain future work, as discussed in Section 6.

**TABLE 2:** Child interaction state detection rules and the robot strategy attached to each state. The derivation of the state set and the provenance of the thresholds are given in Section 3.2.

State	Description	Detection rule	Robot strategy
Silence	Child does not speak after the robot’s question	No speech detected >4 s	Gentle open encouragement
Hesitation	Child responds but lacks confidence to express ideas	Filler words (um, uh, hmm) + word count <15	Easy imagination prompt
Short answer	Child replies briefly without expanding	Word count ≤ 5	Elaboration question
Long answer	Child shares ideas at length but with some uncertainty	Word count ≥ 15 with fillers	Focus and continue
Confidence	Child responds fluently with a rich narrative contribution	Word count ≥ 15, no fillers, fluent	Affirm and advance plot
Repetition	Child repeats a similar idea from the previous turn	Jaccard similarity with previous turn >0.75	Introduce narrative twist
Excitement	Child responds with high energy and enthusiasm	Exclamation marks ≥ 2 or capitalized words ≥ 2	Energetic continuation
Exploration	Child gives a typical answer, not matching any other state	Default, none of the above matched	Open-ended next step

### 2.5 Identified gaps

Across the existing literature, several common limitations can be observed. Early systems developed before the use of LLMs depended on fixed or rule-based content and did not adapt to individual child behavior in real time. After the emergence of LLMs, language generation improved, but most LLM-based storytelling systems still treat the robot as a co-creator rather than supporting the child in leading the storytelling process. These systems often generate story content themselves instead of guiding the child step by step. Interaction states such as silence, hesitation, repetition, and short responses are also rarely addressed in detail, and existing systems do not generate personalized visual outputs from the child’s own story. Studies on visual storytelling show that animation can support creativity and motivation, but these approaches are not typically integrated with interactive robots or adapted during the interaction. Existing systems also rarely provide structured prompting strategies for shy, hesitant, or easily distracted children. The present system is designed to address this gap, although the current evaluation covered only part of this design.

To address these limitations, the proposed system introduces an integrated approach that supports child-led storytelling, adapts to interaction states in real time, and generates a personalized animated story from the interaction. The complete system is implemented and evaluated on real hardware under realistic operating conditions.

## 3 Methodology

This section presents the design and implementation of LLM-Storyteller. Two principles shape the system: the child remains the author of the narrative, and the interaction concludes by rendering that narrative as an animated cartoon.

### 3.1 System architecture overview

The proposed system follows an end-to-end adaptive storytelling pipeline designed to support natural and engaging interaction with children, consisting of two interconnected nodes: a Raspberry Pi five serving as the embodied robot platform, and an MSI laptop equipped with an NVIDIA RTX 4080 GPU acting as the edge computing server. All processing, including speech recognition, language model, text-to-speech synthesis, and cartoon generation, runs locally on the MSI laptop. The Pi and MSI communicate over a local area network. The Pi uploads recorded audio and receives synthesized speech responses. As shown in Figure 1, the system begins when the robot initiates conversation by greeting the child and asking about what topic is needed to generate a story about. The robot will actively listen and wait for speech. Once the child speaks, the Pi records and uploads the child’s audio to the server. On the server, the speech will be converted to text through ASR. The transcribed text then passes through the text input processing layer, which consists of a child interaction state classifier and an adaptive prompt engine (LLM), both of which will be processed in sequence. First, the interaction state extractor analyzes the transcribed text together with timing information from the voice-activity detector, and assigns one of eight storytelling interactions: silence, hesitation, short answer, long answer, confidence, repetition, excitement, or exploration, as shown in Table 2. The state detection follows lightweight heuristic rules. For instance, when the child’s state is flagged as silence, it means that no speech is detected for more than 4 seconds; repetition is identified when the Jaccard similarity between the current and previous turn exceeds 0.75, and excitement is detected through the presence of exclamation markers or capitalized words. As Section 3.2 explains, the last of these rules proved effectively unreachable under the deployed speech front end. Second is the prompt adaptation. Based on the detected state, an adaptive prompt engine selects a guiding question from a fixed prompt bank, which stores several variants per state and draws one uniformly at random. The full bank appears in Listing 2. This prompt is combined with the current story context and passed to a locally deployed language model, Llama 3.2 at 3.2B parameters, served through Ollama under 4-bit quantization (Q4_K_M), which generates a short facilitation question using child-appropriate language. The system is instructed to generate short facilitation questions rather than complete story content, supporting the child’s role as the primary storyteller. The generated response is converted to speech and returned to the robot. The interaction cycle repeats over multiple turns to progressively build the story. Upon completion of the story, the system initiates a story-to-animation pipeline. In the first stage, the session history is filtered to keep only the child’s spoken contributions, while removing all robot prompts, so that the final animation reflects the child’s own story. In the second stage, the filtered story is sent to Llama 3.2 using a structured prompt that extracts five key scenes, where each scene includes an image description and a narration sentence. In the third stage, the image description of each scene is sent to Stable Diffusion XL through the ComfyUI API to generate a cartoon-style image for each scene. In the fourth stage, the narration text for each scene is converted into speech using Edge TTS, producing a WAV audio file for each scene. In the fifth stage, FFmpeg combines the image and audio of each scene into a short video clip, adding simple motion effects to make the static image appear dynamic. In the sixth and final stage, all video clips are combined into a single MP4 file, which is sent to the Raspberry Pi and displayed on the robot’s screen. This allows the child to watch their own story as an animated cartoon, completing the creative feedback loop.

**FIGURE 1:**
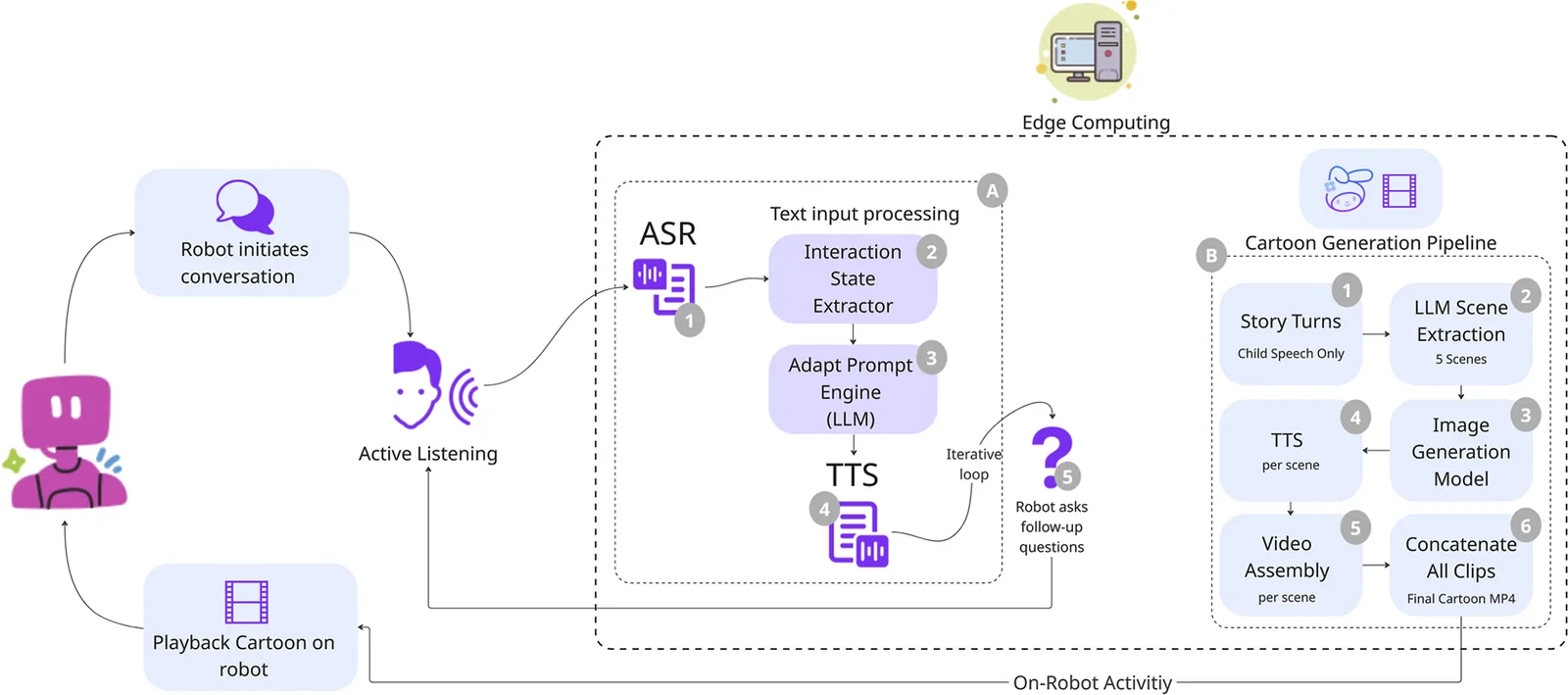
System architecture pipeline for the robot.

### 3.2 Derivation of the eight interaction states

The state set did not come from clustering child speech, and we do not offer it as a validated behavioral taxonomy. It was assembled in two steps. We began with the conversational cues for which the storytelling, tutoring, and child-robot interaction literature provides a general rationale for adapting how a facilitator responds, as reviewed in Section 2.4: silence, brief replies, filled pauses, repeated content, extended speech with or without disfluencies, and high-energy expression. We then sought to retain only cues computable from the interaction transcript and its associated timing metadata, without a vision pipeline, an affect-recognition model, or per-child training data.

These six cues yield seven rule-based states: silence, hesitation, short answer, long answer, confidence, repetition, and excitement. The counts differ because extended speech forks into two states rather than one: a fluent long turn and a long turn containing fillers, which call for different responses from the robot. Exploration was added as an eighth label, a default for transcripts matching none of the other rules. Each state was assigned a lightweight detection rule and a corresponding prompt strategy, and Table 2 reports the resulting mapping.

Three consequences follow from this design. First, the rules are heuristic. Their thresholds, including a five-word ceiling for a short answer, a fifteen-word floor for an extended response, and a Jaccard similarity above 0.75 for repetition, are engineering choices rather than values tuned or validated against child speech. The rules are also applied in a fixed order: silence, repetition, hesitation, short answer, excitement, extended response, and exploration last as the default. The order matters because the rules can overlap and their evaluation order determines the assigned label. A brief utterance carrying a filler is labelled hesitation and not short answer, because the hesitation rule is reached first.

Second, the set is exhaustive only in a computational sense. Every transcript receives exactly one label because exploration absorbs whatever the other rules reject. This is why exploration dominates the distribution in Section 5, and that dominance reflects the classifier design rather than the behavior of the simulated child.

Third, the excitement rule does not in practice satisfy the recoverability criterion that guided the second step. It fires on orthographic markers, two or more exclamation marks, or two or more capitalized words, which belong to the transcript rather than directly to the speech signal. Whisper produces punctuation and sentence-initial capitalization, but rendering vocal emphasis as exclamation marks, or a raised voice as capitalized words, is not behavior on which a detector can rely. Across the 72 child turns in our sessions, it produced no exclamation marks, and the only all-capitals token in the corpus was a transcription artifact rather than an expression of enthusiasm. The rule was therefore effectively unreachable under the deployed ASR configuration. Its absence from the distribution in Section 5 should be interpreted accordingly: the state received no positive empirical test case, and that absence cannot be attributed to the simulated child’s behavior alone. Silence, by contrast, reaches the classifier through timing metadata that the pipeline does provide, which is exactly what excitement lacks. Detecting enthusiasm in speech would require prosodic features such as pitch and energy, extracted from the audio before this information is largely lost or left unrepresented in the transcript. Section 6 therefore treats this as a correction required by the pipeline rather than a capability already provided by it.

### 3.3 Prompt templates

Three artifacts drive the language side of the system, and all three are reproduced here so that the pipeline can be replicated. The first is the system prompt sent to Llama 3.2 on every dialogue turn. It carries the story context and the constraints that keep the robot in a facilitator role, and from turn four onward a wrap-up instruction is appended so that the story reaches an ending rather than drifting.

The system was originally designed for children aged 5 to 10. During the simulated sessions, the short sentences and simple vocabulary produced by the system also appeared suitable for the broader 5 to 12 age range used in the role-play. The prompt reproduced here retains the original 5 to 10 range because it is shown exactly as deployed during the logged sessions. This difference concerns only the requested language register and does not affect the pipeline or interaction-state logic. The broader age range remains to be evaluated with children.

The second artifact is the prompt bank, which fills the adaptive_prompt placeholder above. It is a fixed set of guiding questions stored per state, and one variant is drawn uniformly at random from the entry for the detected state, so a state that recurs across turns does not produce an identical robot question. The bank is reproduced in full in Listing 2. Most prompts ask the child to elaborate on previously introduced content or to provide the next story element. This question-based design is consistent with the facilitative role examined through the authorship indicators in Section 4.4.

The third artifact is the scene extraction call, issued once the story is complete. It receives the filtered child-only narrative and returns five scenes as JSON, each with an image description and a narration sentence.

### 3.4 Content safety

The system has no dedicated safety classifier. What it has is a pair of fixed prompt constraints applied to every image generated by SDXL. A positive style prefix requests a 2D children’s cartoon rendered in the visual register of major animation studios, and a negative prompt blocks a fixed list of undesired content, namely, realistic, photo, dark, scary, violence, adult, text, watermark, blurry, low quality, and 3D render. Both strings are constant across all sessions and all five scenes, and they operate before generation rather than after it. This is a rule-based constraint, not a filter: nothing inspects the generated image, and nothing inspects the child’s speech or the model’s follow-up question for unsafe content. A deployment with real children would need a post-generation classifier on the image and a moderation pass on the dialogue, and Section 6 carries this as an explicit gap rather than a solved problem.

Listing 1. **Prompt templates for one dialogue turn, reproduced from the deployed system.**

— SYSTEM PROMPT —

You are a friendly, warm, and encouraging storytelling robot assistant for children aged 5–10.

You help children BUILD their own stories step by step - you never write the story for them.

Your role is to ask ONE short, imaginative question that helps the child continue their story.

Current story: “{title}”

Characters so far: {char_str}

Story summary so far: { summary, or “The story is just beginning.” }

Turn number: { turn }

Rules:

- Ask ONE question only. Never write paragraphs.

- Keep your response under 2 sentences.

- Be warm, enthusiastic, and encouraging.

- Use the child’s own words and ideas.

- Never introduce plot elements.

- only ask questions that help the child invent them.

- If the story seems complete, gently suggest wrapping up.

- Simple language suitable for children.

[appended when turn >= 4]

This is the last turn. Ask for a wrap-up to the story to prepare for the proper story ending.

— USER MESSAGE —

The child said: “{ child_text }”

{adaptive_prompt}

Listing 2. **The complete prompt bank. One variant is selected uniformly at random from the entry matching the detected interaction state.**

silence:

 “That is okay! Take your time. What is one thing you see in your story?”

 “No rush! Can you tell me one thing about your character?”

 “It is okay to think slowly. What color is something in your story?”

hesitation:

 “You are doing great! What is the first thing that comes to your mind?”

 “There is no wrong answer! What does your character want to do?”

 “Even a tiny idea is perfect! What does your character see right now?”

short_answer:

 “Oh I love it! What happens after that?”

 “Wow! And then what does your character do?”

 “Great! Where does this happen?”

 “Cool! What does it look like there?”

repetition:

 “Yes! And now something surprising happens - what is it?”

 “I love that part! What if a new friend shows up?”

 “Amazing! What happens next that nobody expects?”

excitement:

 “Yes yes yes! Keep going, what happens next?!”

 “I love your energy! What does your character do now?”

 “So exciting! What is the most fun part coming up?”

exploration:

 “Ooh interesting! What does your character find?”

 “I like it! What does your character want most right now?”

 “Great thinking! What is the biggest problem your hero has to solve?”

confidence:

 “You are an amazing storyteller! How does the story end?”

 “Wow so good! How does your character change at the end?”

 “Brilliant! What is the message of your story?”

long_answer:

 “You have such a big imagination! What is the most exciting part?”

 “I love your story! What does your character decide to do?”

 “So good! How does the adventure continue?”

### 3.5 Hardware components & design

The proposed robot requires a set of hardware components, including a Raspberry Pi five to connect all the hardware components, a microphone to detect the voice and convert it to speech, a speaker to hear the robot’s response, and a screen to show the interaction face and display the generated cartoon. The robot features a child-friendly design and is printed using PLA material, as shown in Figure 2.

**FIGURE 2:**
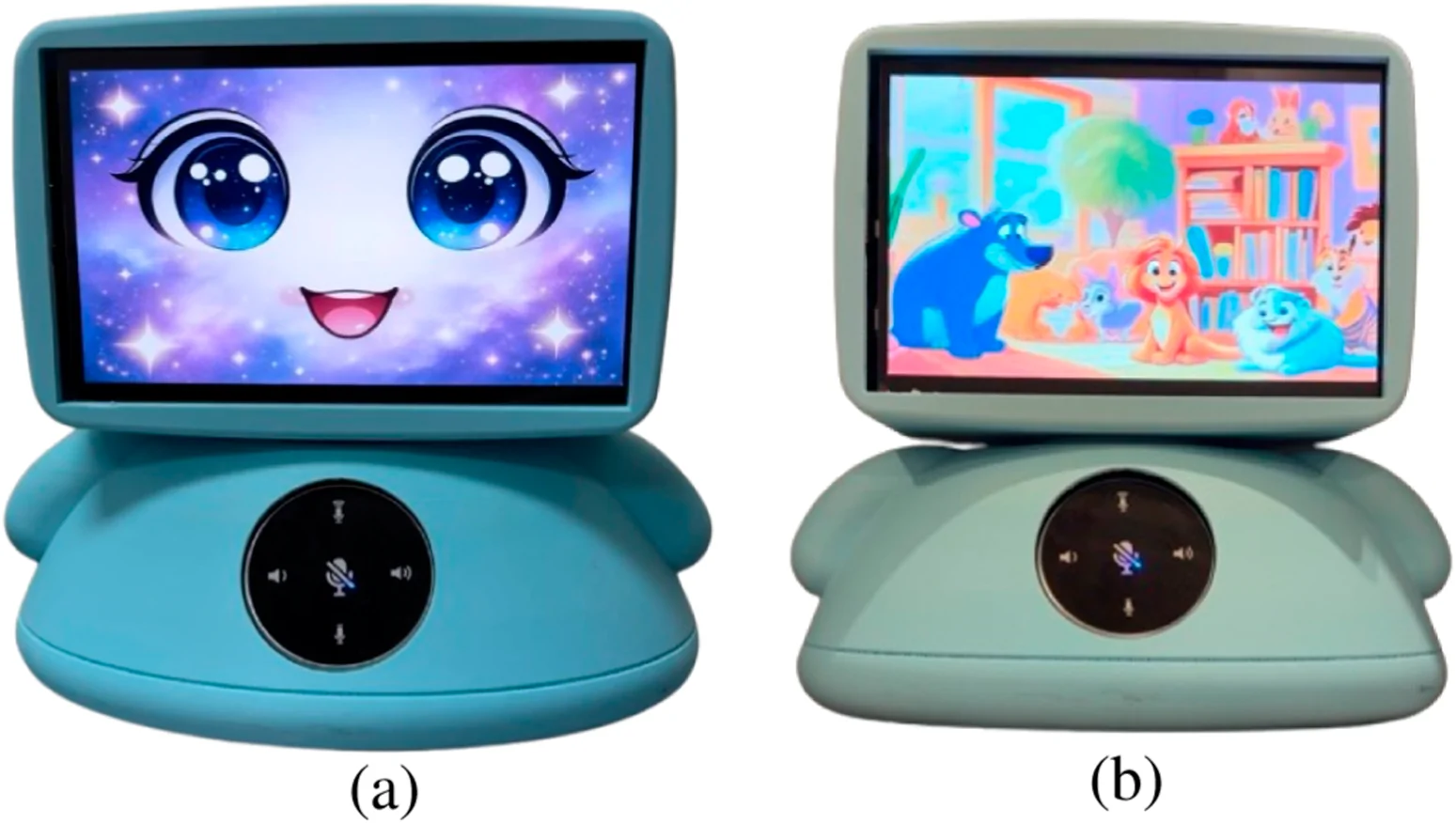
Embodied storyteller robot. **(a)** Robot interaction mode. **(b)** Generated story display.

## 4 Evaluation

This section presents the implementation, the system performance metrics, the simulation protocol, and the internal rating procedure.

### 4.1 Implementation details

This section presents the tools and models selected to implement the robot system shown in Table 3. The robot platform is the Raspberry Pi 5, and the language used is Python 3.11. The server is an MSI laptop running Windows 11 with CUDA 12.8. All software components are open-source. The ASR used is Whisper-based, with 74M parameters (Radford et al., 2023), and TTS is Microsoft Edge with the voice model “en-US-AvaNeural”. The LLM is Llama 3.2 (Grattafiori et al., 2024) served locally through Ollama at 3.2B parameters with 4-bit quantization, Q4_K_M, and a 131,072-token context window. Follow-up questions are generated at temperature 0.85 with an 80-token cap, while the scene extraction call that feeds the animation pipeline runs at the lower temperature of 0.4. The image generation pipeline uses the Stable Diffusion XL (Podell et al., 2023) base model sd_xl_base_1.0.safetensors accessed via the ComfyUI REST API, with a fixed sampler configuration of 25 denoising steps, CFG scale 8, Euler sampler, and normal scheduler. Images are generated at 768 × 512 pixels. In the story-to-animation pipeline, a fixed cartoon style prefix is appended to every SDXL prompt to ensure visual consistency across all five scenes of each session, together with a fixed negative prompt described in Section 3.4. The video assembly pipeline applies a Ken Burns pan-and-zoom effect using the FFmpeg zoompan filter, including a zoom range of 1.0–1.2 over the clip duration at 25 fps, producing MP4 files compatible with the Raspberry Pi VLC player.

**TABLE 3:** Software models and frameworks. Configuration values used in the 12 logged sessions.

Component	Model/Framework	Version/Configuration
Interaction loop		
Speech recognition	OpenAI whisper	Base, 74M parameters, fp16 disabled
Language model	Meta llama 3.2 via ollama	3.2B, Q4_K_M, 131k context, T = 0.85, 80-token cap
Text-to-speech	Microsoft edge TTS	en-US-AvaNeural
Robot API server	FastAPI + uvicorn	Python 3.11, port 8000
HTTP communication	Httpx client, FastAPI server	Multipart POST over LAN
Story-to-animation pipeline		
Scene extraction	Meta llama 3.2 via ollama	T = 0.4, five scenes as JSON
Image generation	Stable diffusion XL	sd_xl_base_1.0, 25 steps, CFG 8, euler
Generation interface	ComfyUI	REST API, local server, 768 × 512
Video assembly	FFmpeg	H.264, yuv420p, ken burns zoompan, 25 fps
Robot platform		
Face animation	Pygame	v2.6.1, PNG layers over HDMI
Video playback	VLC (cvlc)	HDMI fullscreen, USB audio
Session logging	SQLite + openpyxl	Per-turn logs, excel export

### 4.2 System performance metrics

To ensure that the system works under realistic conditions, 12 interaction sessions were conducted with the robot to evaluate the response latency and cartoon generation time. Latency is defined as the time from when the Pi finishes sending the audio to when it starts playing the response, including measures of Whisper transcription, child state classification, LLM inference, and Edge TTS synthesis from the server side. In addition, the story-to-animation pipeline measures the following separately: LLM scene extraction, SDXL image generation, Edge TTS narration, FFmpeg video assembly, and FFmpeg concatenation, then calculates the average. Table 4 shows the average latency of response and cartoon generation time. State distribution frequency is measured during each turn, ensuring the coverage of the state detection classifier in real interaction conditions, as shown in Table 5.

**TABLE 4:** Response latency and cartoon generation time across the 12 sessions.

Component	Mean ± sd	Min – max
Response latency (s)	1.08 ± 0.55	0.64–2.23
Cartoon generation time (s)	79.3 ± 44.5	51.3–182.8

**TABLE 5:** Child interaction State distribution across the logged Sessions.

Detected state	Occurrences	%
Exploration	37	51.4
Confidence	26	36.1
Hesitation	5	6.9
Short answer	3	4.2
Long answer	1	1.4
Silence	0	0.0
Repetition	0	0.0
Excitement	0	0.0
Total	72	100.0

### 4.3 Simulation protocol and data provenance

The validity of everything that follows depends on where the child utterances came from, so we describe their origin in full. The evaluation consists of 12 simulated storytelling sessions. The two authors conducted six sessions each, working independently. In every session, one author role-played a child using language and imagination considered appropriate for the general target range, without assigning a specific age to the session. No large language model produced the child side of the dialogue, no external dataset was used, and no session followed a script.

Each session opened with a story topic chosen freely by the author playing the child, drawn from ordinary childhood territory such as animals, adventure, friendship, fantasy, and everyday life. The story was then built turn by turn. The author spoke into the microphone, the system replied through the pipeline described in Section 3.1, and the author answered the question it produced. This continued until the story reached an ending. Every turn passed through the full pipeline, including the ASR stage, rather than being injected as text. No scenario design criteria were imposed. The intention was to test whether the robot’s adaptive guidance could keep a story moving whatever the opening topic, and whether it could draw more detail out of the speaker rather than let the narrative stall, so constraining the topics would have defeated the purpose. No age was assigned to any individual session. The 5 to 12 band is our own judgment, based on the register the system produced, short sentences and plain vocabulary, rather than on any measurement, and no session was played at a specific age within it. The data therefore cannot support an analysis of how performance varies with the child’s age. That limitation is stated again in Section 6.

After a session ended, the system automatically kept only the child-side utterances, reorganized them into a continuous narrative, extracted five scenes, and produced the narrated cartoon. These steps altered nothing in the interaction itself. No manual editing, correction, filtering, or curation was applied to the ASR transcripts or to the dialogue at any point before rating. The outputs were assessed exactly as the system produced them, and all 12 transcripts are released as supplementary material.

The sessions were cooperative and fluent by design because the evaluation focused on whether the robot’s adaptive questions could sustain the storytelling process and support the development of a complete story. This design did not exercise all interaction states. Silence and repetition were not observed in the sessions, while excitement was effectively unreachable because its detector relied on orthographic markers that the ASR front end did not produce. The detection and prompting machinery therefore operated on the five states that occurred.

### 4.4 Approximating narrative authorship: a lexical-origin measure

RQ1 asks whether the robot facilitates without taking over the story. None of the four rating dimensions captures who supplied the narrative vocabulary, so we define two indicators computed directly from the session transcripts. Both provide limited transcript-level evidence about the robot’s facilitative role. Neither is a semantic measure of narrative ownership, and neither establishes who determined the events or direction of the story.

Lexical Narrative Ownership (LNO) is the fraction of the unique content words in the finished narrative that the child uttered before the robot did. For each session, the turns are tokenized in chronological order, function words and discourse markers are removed using a fixed stop list, and every remaining word is attributed to whichever speaker produced it first. The narrative is the set of content words appearing in the child’s turns, since the animation pipeline is driven by the child-only text. LNO is then calculated using Equation 1:

$$LNO=\frac{\left|\left\{w\in C\;:\;\mathrm{fi}rst\left(w\right)=\text{child}\right\}\right|}{\left|C\right|}$$ (1)

where C is the set of unique content words in the child’s narrative. A value of 1.0 means that every unique content word in that set was first produced by the child. A lower value means that a larger share of the vocabulary had already appeared in a robot turn.

Question compliance is the proportion of robot turns that contain at least one question mark and no more than two sentence-like segments, where the segments are obtained by splitting the response at periods, exclamation marks, and question marks. It measures surface-level conformity to the question-based response format. It does not determine whether a turn contains exactly one semantic question or whether a question introduces a new plot event.

The distinction between lexical origin and semantic ownership can be illustrated with an example. Suppose the child introduces a dragon and a king, after which the robot asks what happens when they meet. Words first produced in the child’s answer are attributed to the child, although the meeting itself was suggested by the robot. LNO does not capture this form of narrative guidance, and question compliance does not assess the semantic contribution contained within the question.

Both indicators should therefore be interpreted as lexical and structural proxies computed from adult role-play transcripts rather than child speech. They provide reproducible descriptions of the observed interaction but do not by themselves demonstrate that every narrative decision originated with the child. Future work could address this limitation by annotating each story event according to whether it was introduced by the child or the robot.

### 4.5 Internal rating of system outputs

The quality of the language and visual outputs was assessed by the two authors of this paper. We next describe the evaluation procedure and the scope of the resulting findings.

Both raters have a background in child-robot interaction. Each independently rated all 12 sessions on four dimensions using a five-point rating scale. The evaluation material consisted of the complete child-robot dialogue transcript and the generated cartoon video. Agreement was assessed using linear-weighted Cohen’s kappa (Cohen, 1968) and mean absolute deviation. The value reported for each session and dimension is the mean of the two independent scores.

The outputs were rated by the two authors who developed the system, so the evaluation was not independent and may be subject to confirmation bias. The small number of raters also limits the generalizability of the subjective judgments. We retain these ratings as a descriptive assessment of the generated outputs. The results should therefore be interpreted as preliminary developer-based judgments rather than as an independently validated measure of output quality.

#### 4.5.1 Evaluation dimensions

Four dimensions were used, and Table 6 defines them. These dimensions were developed specifically for this system rather than adopted from a validated instrument. Two assess the language outputs, and two assess the visual outputs, with the rating anchors defined before the evaluation began. None of the four dimensions measures narrative authorship; this is instead examined using the transcript-derived indicators described in Section 4.4. The dimensions are therefore treated as study-specific proxies for output quality.

**TABLE 6:** Rating dimensions and scoring rubric.

Dimension	What is rated	Scoring criteria	Evaluation level
D1: Follow-up question quality	Does the robot question help the child continue the story? Is it child-friendly and contextually relevant?	5 = perfectly appropriate, directly builds on child’s words 4 = good but slightly generic 3 = acceptable, loosely connected 2 = off-topic or confusing for a child 1 = makes no sense in context	Per session
D2: Story coherence	Does the story make sense across all turns? Does the LLM maintain context and character consistency?	5 = flows naturally, characters consistent, events connect logically 4 = mostly coherent with minor issues 3 = some coherence, occasional context loss 2 = fragmented, characters shift without reason 1 = No logical connection between turns	Per session
D3: Image–story alignment	Does the generated cartoon image match the scene described in the child’s story?	5 = image clearly depicts the scene including characters, setting and mood 4 = mostly matches with minor differences 3 = partially related, misses key elements 2 = loosely related to the scene 1 = No connection to the story	Per session
D4: Image quality	Is the image visually appropriate for a child, cartoon style, bright colors, not scary or inappropriate?	5 = clear cartoon style, bright, child-friendly, high quality 4 = good quality with minor issues 3 = acceptable but not strongly cartoon-style 2 = poor quality or not appropriate for children 1 = completely inappropriate or very low quality	Per session

## 5 Results and discussion

The evaluation was conducted at multiple levels: system performance metrics, child state detection, narrative authorship, and an internal rating of language and visual outputs across 12 simulated sessions.

### 5.1 System performance

Table 4 reports the response latency and cartoon generation time measured across all 12 sessions. The average response latency was 1.08 s ( ± 0.55 s), with a range from 0.637 s to 2.233 s. The variation likely reflects differences in processing time across the response-generation pipeline, including LLM inference. Eleven of the twelve sessions stayed under 2 s. The single exception reached 2.233 s. For cartoon generation, the average was 79.3 s ( ± 44.5 s) across all sessions, with two sessions requiring the longest time (161.2 s and 182.8 s), due to more complex scene content that increased SDXL inference time. The rest of the sessions completed within 51 s–88 s, which is a reasonable range given that cartoon generation runs after the interactive part ends.

Regarding child interaction state detection, 72 turns were logged across the 12 sessions, six per session, comprising the opening turn in which the simulated child names the topic and the five turns that build the story. Table 5 gives the full distribution. The most frequent state was exploration at 51.4% of turns (37), which is the default label assigned when no other rule fires and therefore carries no behavioral meaning of its own. Confidence followed at 36.1% (26 turns), meaning the utterance was fluent, at least fifteen words long, and free of filler words. Hesitation appeared in 6.9% of turns (5), short answer in 4.2% (3), and long answer in 1.4% (1). Silence, repetition, and excitement were not observed, leaving five of the eight states with positive test cases. Sections 3.2 and 4.3 explain the reasons.

### 5.2 Narrative authorship

Table 7 reports the two authorship indicators defined in Section 4.4. Across the 12 sessions, 272 of the 313 unique content words in the simulated child’s narrative appeared first in a child turn, giving a mean Lexical Narrative Ownership of 0.87 ( ± 0.06), with a range from 0.76 in S1 and S4 to 0.96 in S2. Robot turns followed the measured question-based response format in 58 of 72 cases. Of the fourteen exceptions, eight occurred at turn four or later, where the system prompt switches to a wrap-up instruction and a closing statement is the intended behavior. The other six occurred earlier in the interaction and did not meet the measured response-format criterion.

**TABLE 7:** Per-session lexical narrative ownership and question compliance.

Session	Unique content words	Child-first	LNO	Question compliance
S1	17	13	0.76	5/6
S2	23	22	0.96	6/6
S3	29	27	0.93	6/6
S4	21	16	0.76	6/6
S5	17	15	0.88	6/6
S6	22	19	0.86	5/6
S7	22	19	0.86	3/6
S8	28	24	0.86	4/6
S9	37	32	0.86	4/6
S10	33	29	0.88	5/6
S11	37	31	0.84	3/6
S12	27	25	0.93	5/6
All	313	272	0.87 ± 0.06	58/72

The 41 content words that entered the narrative from the robot are worth naming, because they show where the influence actually lies. They are dominated by affective and relational vocabulary, words such as magical, happy, excited, sad, worried, friend, and secret, with a small number of concrete nouns such as key, alien, bear, school, and wing. In other words, the robot’s questions supplied emotional framing that the simulated child then took up, while the entities and settings of each story originated on the child’s side. This is consistent with the design intent, which forbids the model from introducing plot elements but not from asking how a character feels.

Two caveats bound these numbers. The measure is lexical, so it records who first said a word rather than who decided what happens, and a robot could in principle steer a plot without introducing a single new content word. The narratives are also adult role-play, and an adult playing a child is likely to be more lexically productive and less suggestible than a child would be. Both indicators should therefore be read as an upper bound on the authorship that the system would preserve with real children, not as an estimate of it.

### 5.3 Internal rating results

Table 8 presents the rating results across the four dimensions, averaged over both raters and all 12 sessions. The combined session average was 16.08 out of 20, with Rater one averaging 16.83 and Rater two averaging 15.33. Figure 3 shows the per-session total scores for both raters across all 12 sessions.

**TABLE 8:** Internal rating results averaged across two raters and 12 sessions.

Dimension	Mean/5	SD	Min – Max	MAD	Weighted κ
D1: Follow-up question quality	4.42	0.29	4.0–5.0	1.17	n/a
D2: Story coherence	4.17	0.65	3.0–5.0	1.00	−0.04
D3: Image–story alignment	3.54	1.01	1.5–5.0	0.58	0.49
D4: Image quality	3.96	0.86	3.0–5.0	0.42	0.57
Session total/20	16.08	1.82	13.5–19.0	—	—

**FIGURE 3:**
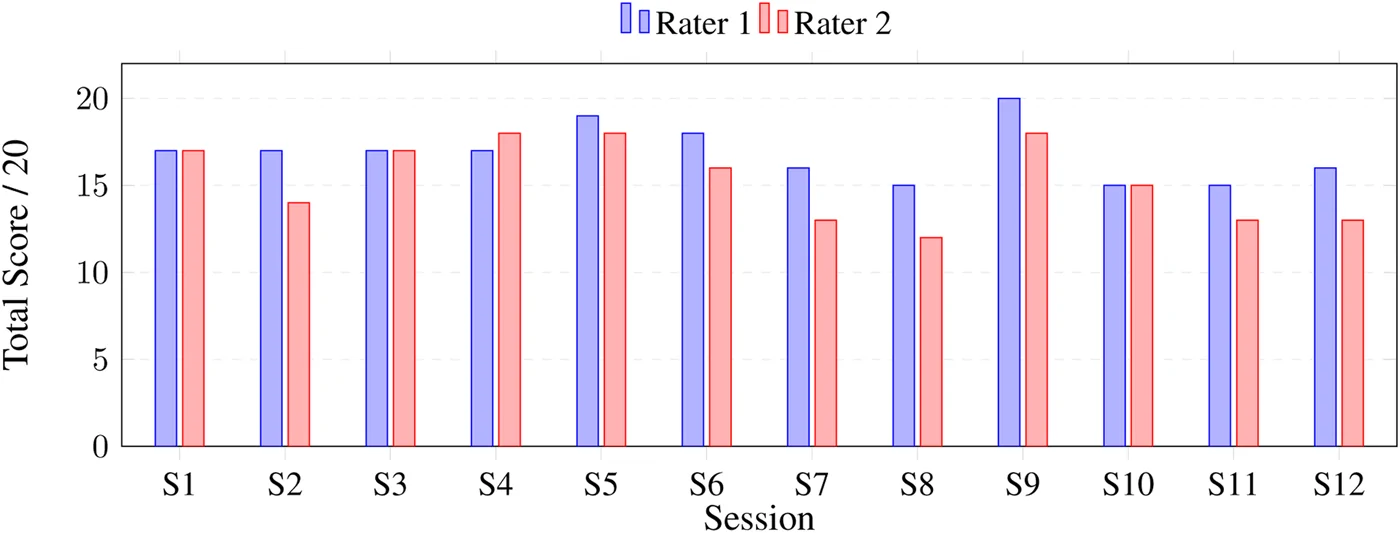
Per-session total scores for Rater one and Rater two across 12 sessions.

D1: Follow-up Question Quality scored the highest among all dimensions, with a mean of 4.42 ( ± 0.29) and all session averages falling between 4.0 and 5.0. Rater one assigned a score of five to all 12 sessions, whereas Rater 2’s scores ranged from three to 5, giving a MAD of 1.17. Weighted kappa is not informative here. Rater one used a single score category, so there is no variation in that rater’s judgments for kappa to work with, and Table 8 reports it as not applicable. The MAD is the more direct measure of the distance between the two raters. The rating comments describe the questions as relevant to the child’s preceding response and appropriate for the target age group, which is what the high D1 scores reflect.

D2: Story Coherence achieved a mean of 4.17 ( ± 0.65), indicating that the LLM maintained narrative flow across turns in most sessions. The MAD of 1.00 and kappa of −0.04 indicate differences in how the two raters applied the coherence criteria. With twelve items and two raters, kappa is estimated from a small sample, so these values are treated as descriptive rather than as evidence of a particular level of agreement. The rating comments record the same problem across several sessions: the LLM changed the gender of the main character mid-session without any instruction from the child. This contributed to the lower coherence scores.

D3: Image Story Alignment had the lowest mean of 3.54 ( ± 1.01) and the widest score range, from 1.5 in S8 to 5.0 in S9. The kappa of 0.49 indicates moderate agreement between raters on the conventional scale (Landis and Koch, 1977), with a MAD of 0.58. The evaluator comments pointed to two main issues. First, character appearance was inconsistent across the five generated images within the same session, as SDXL does not preserve character identity between separate inference calls. Second, objects that played a key role in the child’s story were sometimes missing from the generated images. For example, in one session, a magical pen that the child referenced throughout was absent from all five images. In another, a princess character introduced at the start was replaced by a boy in later images.

D4: Image Quality achieved a mean of 3.96 ( ± 0.86), with the highest inter-rater agreement among the four dimensions, reflected by a kappa of 0.57 and a MAD of 0.42. Images receiving the highest scores were described as having a clear cartoon style, bright colors, and no visible artifacts. The five sessions with an average score of 3.0 received a similar comment from the raters: the visual style appeared more suitable for teenagers than for young children. No session scored below 3.0, and none of the 60 generated images was rated as unsafe or inappropriate.

### 5.4 Answers to the research questions

Answer to RQ1. Most of the narrative vocabulary originated with the simulated child rather than the robot, based on the two transcript indicators reported in Section 4.4 and Table 7. These findings describe lexical origin and response structure, not semantic ownership of individual story events.

Answer to RQ2. The integrated system operated successfully on the evaluated edge setup, with the performance, interaction-state coverage, and internal rating results reported in Sections 5.1 and 5.3 and in Tables 4, 6, 8. The results support the technical feasibility of the prototype under the evaluated conditions.

### 5.5 Discussion

The results show where the system performs well and where the pipeline needs refinement. Follow-up question generation was the strongest component. The robot’s questions generally remained connected to what the simulated child had just said and used language suited to the target range. The transcript-based authorship indicators support this reading without relying on the internal ratings. Restricting the LLM to questions rather than story content appears to help keep the narrative development on the child’s side.

Story coherence was affected by recurring character-identity drift across some sessions. Inspection of the logs suggests that the character-tracking heuristic contributed to this problem. The heuristic stores capitalized words longer than two characters after applying a short stop list. Since Whisper capitalizes the first word of each transcribed sentence, discourse markers and pronouns were sometimes stored as character names. Entries such as Yes, Actually, Once, and There appeared in the stored list, and the pronoun She was recorded as a character in seven sessions. These entries were then inserted into later prompts, which provides a plausible explanation for part of the gender drift noted by the raters.

Image-story alignment was affected by two parts of the animation pipeline. SDXL generates each image independently from its scene prompt, so the same character can appear differently across the five scenes. The scene-extraction step can also omit objects that seem minor in a summary but remain important to the story.

The comments on age appropriateness point to the fixed style prefix, which was applied across all sessions, rather than to the content of any individual story.

The timing results indicate that the dialogue pipeline is suitable for conversational use on the evaluated edge setup, while cartoon generation is better treated as a post-interaction stage. Longer scene descriptions appear to increase image-generation time, so controlling prompt length may reduce the waiting period without changing the underlying model.

The state distribution reflects both the classifier design and the scope of the simulated sessions. Exploration appears frequently because it is the default label when no other rule applies. The sessions exercised only part of the eight-state design, and the implications of the uncovered states are discussed in Section 6.

## 6 Limitations and future work

The main limitation of this study is that no children participated. All 12 sessions were produced by the two authors role-playing children within the target age range of 5–12 years. Adult role-play may not reflect child speech patterns such as hesitation, brevity, repetition, topic drift, or sudden enthusiasm. The findings therefore apply only to the simulated conditions. The authorship indicators in Section 4.4 should also be read as measures of lexical origin rather than semantic story ownership. They do not show who introduced each narrative event, which would require turn-by-turn semantic annotation.

The output ratings were also conducted by the two authors who developed the system and were based on only 12 sessions. Section 4.5 explains how this affects the interpretation of the ratings and agreement statistics.

The simulated sessions covered only part of the eight-state design. Silence and repetition were not observed, while excitement was not recoverable under the deployed ASR configuration. The sessions were also not assigned specific child ages, so the present data cannot show whether system performance varies across the 5 to 12 age range.

The current safety mechanism is limited to a fixed style prefix and negative prompt applied before image generation. The pipeline does not yet include post-generation image screening or separate moderation of the child’s speech and the robot’s responses. Although no unsafe content was observed in the 60 generated images, the sessions used benign topics and do not provide broad evidence of safety.

The prototype also showed limitations in character tracking and image consistency. Character identity sometimes drifted across turns, while SDXL occasionally changed character appearance or omitted important story objects across scenes. The technical causes are discussed in Section 5.5.

Future work will evaluate the system with children and independent raters, using measures of recall, causal understanding, and character tracking alongside engagement. Expert-informed scenarios will be used to improve coverage of the interaction states. The next system revision will also add post-generation image screening, dialogue moderation, more reliable character extraction, validation of scene prompts against the child’s original story, and character-preserving image-generation methods.

## 7 Conclusion

In this paper, we presented LLM-Storyteller, an adaptive embodied robot designed to facilitate child-led narrative co-creation and personalized story visualization. By combining real-time interaction state detection with an adaptive prompt engine, the robot guides the storytelling process while leaving the narrative in the child’s hands. The integration of a local story-to-animation pipeline closes the loop by turning the spoken narrative into a multimedia cartoon.

The evaluation shows that the system can support child-guided storytelling as an integrated prototype, combining adaptive dialogue with local story-to-animation generation. The transcript-based analysis indicates that the simulated child supplied most of the narrative vocabulary, while the internal ratings point to stronger language output than visual alignment.

The findings are limited to simulated sessions. Future work should evaluate the system with children and independent raters, extend coverage of the interaction states, and improve excitement detection through prosodic features. These steps are needed before the system can be assessed for use in practice.

The work advances UN SDGs related to quality education (SDG 4) and innovative infrastructure (SDG 9).

## Data Availability

The materials are available in a public repository: https://github.com/fatimah48/LLM-Storyteller. The repository contains the 12 session transcripts, prompt bank, authorship-analysis script, rating sheet, and two robot recordings.
